# Contagion of Plague—Dr. Granville *versus* the Late Dr. Gooch

**Published:** 1831-01-01

**Authors:** 


### XXII.

### CONTAGION OF PLAGUE.

THE public and the profession are aware that the late Dr. Gooch wrote an elaborate article in the Quarterly Review on the subject of plague and of quarantines, which he subsequently re-published in his work on the diseases of females, with his name appended. In some prefatory observations to this re-publication, he makes use of the following words:—

"About the year 1825 great pains were taken to persuade the legislature, that the plague is not a contagious disease, and that the *quarantine* laws are a useless burthen to trade. Two committees were appointed by parliament to hear evidence on the subject, and the government was on the eve of modifying and relaxing those laws. There were many persons far more competent than myself to put the question in its true light; but seeing that *not one would undertake it,* I wrote the following paper, and published it in the Quarterly Review, as the best pulpit from which to address the government and the people of England." . . . "The article worked well. I received a communication from his Majesty's ministers that it satisfied their minds on the subject; *the quarantine laws were left untouched;* and the subject which had been so actively discussed before, has been since scarcely alluded to."—(Gooch, Appendix to the diseases of Females, 1829.)

Upon this paper Dr. Granville has made a pertinent commentary in our cotemporary, the Medical Gazette—and we only wonder that he should have so long permitted the passage to stand uncriticised. It would have been better had he published these remarks before the death of the author of the review—though certainly it is not too late. We recollect the parliamentary enquiry which then took place, for we were one of those who were examined on the occasion, and we think Dr. Gooch gave himself a great deal too much credit in supposing that it was *his* article in the QUARTERLY, which turned the scale against the anti-contagionists of plague—that it was "his thunder" which shook Dr. Maclean and his disciples from their throne—and not the evidence brought forward by the committee. We differ widely from him on this point. Dr. Granville underwent a long examination on that occasion, and published a pamphlet in which he collected a great number of historical facts blended with his own personal experience. It is but fair to infer that his viva voce evidence and his written statements contributed materially to the quashing of the wild speculations at that time broached by Dr. Maclean, and believed by a considerable number of non-professional persons.

Dr. G. avers that Dr. Gooch, as well as the editor of his life, was wrong in dates—wrong as to facts—and wrong as to conclusions.

"It was in 1819, and not in 1825, that 'great pains were taken to persuade the legislature that the plague was not a contagious disease.' The immediate effect of this attempt on the part of the anti-contagionists was the appointment of a Committee, in 1819, followed by a Report to the House of Commons, in which it was stated, that the opinion of all the competent witnesses who had been examined, except two, *had proved to the satisfaction of the Committee*, that the plague was a contagious disease: and that report so far settled the question of contagion to the satisfaction of the King's Ministers, that not another word was uttered on the subject for five years after, nor any attempt made to disturb the quarantine laws during that period. Was Dr. Gooch examined before that Committee? Was he deemed a competent witness? No; his name does not even appear; whereas mine stands not unconspicuously among those of several contagionists who brought forward a host of facts in support of their doctrine. But as mere oral evidence appeared to me insufficient on that occasion, I undertook, in a work written for the purpose that same year, to answer all the objections started by the apostle of non-contagion, Dr. Maclean. I disproved in that work all his assertions, and brought forward so many additional facts, of several of which I had been an eye-witness, that none but Dr. Gooch ever ventured subsequently to say 'that no competent person *before him* had undertaken to put the question in its true light.' Dr. Gooch compiled his article in the Quarterly with my own arguments, my own references, my own quotations, and narrative of events; and, above all, with the principal facts contained in my letter on the plague and contagion, addressed to the President of the Board of Trade, in 1819, (Lord Goderich.) He also borrowed largely (and still without acknowledgement) from the printed evidence laid before the House of Commons, which went far to shew 'the question in its true light.' Nay, the very words with which the Report of that memorable Committee conclude afford a striking proof that the question of contagion required not the farther assistance of a purely theoretical writer on the plague like Dr. Gooch, in order to be settled. 'Your Committee *see no reason* to question the validity of the principles on which quarantine regulations appear to have been adopted.'

The above passage, and various other statements in Dr. Granville's paper, shew that Dr. Gooch assumed a great deal too much, and that, like some other writers in the QUARTERLY, his brain was turned by the smiles and the gold of Mr. Murray.

---

